# Complete genome sequence of a *Listeria monocytogenes* strain isolated from an imported product of enoki mushroom

**DOI:** 10.1128/mra.00180-25

**Published:** 2025-07-09

**Authors:** Calvin Ho-Fung Lau, Mathu Malar C., Liam P. Brown, Lang Yao, Bridgette Kelly, Chloe Anastasiadis, Lenka Lukic

**Affiliations:** 1 Ottawa Laboratory (Carling), Canadian Food Inspection Agency 5737, Ottawa, Ontario, Canada; The University of Arizona, Tucson, Arizona, USA

**Keywords:** enoki mushroom, *Listeria monocytogenes*, food safety, bacterial genomics

## Abstract

Here, we report the complete genome sequences of a *Listeria monocytogenes* strain isolated from an imported retail product of enoki mushroom in Canada. The genome consists of a single circular chromosome (2,994,174 bp) and a plasmid (86,632 bp) with a total guanine-cytosine content of 37.9 %.

## ANNOUNCEMENT

The gram-positive facultative anaerobic bacterium *Listeria monocytogenes* is a significant foodborne pathogen and the primary causative agent of listeriosis—a disease that can pose a serious health risk to the elderly, infants, pregnant women, and the immunocompromised (1). This report documents and describes the complete genome sequence of an *L. monocytogenes* strain (designation CFIAFB20240043) recovered from an imported enoki mushroom product purchased in Ottawa, Ontario, Canada, in 2024. The strain was originally recovered from 25 grams of the enoki mushroom sample that was aseptically transferred into a stomacher bag containing 225 mL of BD Difco UVM-modified *Listeria* Enrichment Broth (Thermo Fisher Scientific, Canada). After homogenization, the enoki-containing bag was incubated for 24 h at 30°C without agitation, followed by *L. monocytogenes* isolation using RAPID *L.mono* medium agar (BioRad, Canada) with incubation at 37°C for 24 h. The identity of the isolate was subsequently confirmed as *L. monocytogenes* by hemolysis test on blood agar plates (35°C, 24 h), fermentation test on carbohydrate (mannitol, rhamnose, and xylose) agar plates (35°C, 24 h), and based on motility observation using a phase contrast microscope.

Genomic DNA was extracted separately from two identical overnight cultures of *L. monocytogenes* CFIAFB20240043, prepared by inoculating a single colony (revived from a frozen glycerol stock of the original isolate) in brain heart infusion medium (Oxoid, Canada), using a Maxwell 16 cell DNA purification kit (Promega, USA) for Illumina sequencing and a Qiagen DNeasy Blood & Tissue kit (Qiagen, Canada) for nanopore sequencing, with quantification using a Qubit dsDNA HS Assay Kit (Thermo Fisher Scientific, Canada). Illumina sequencing was carried out by library construction using a Nextera XT library prep kit (Illumina, USA), followed by paired-end sequencing using a MiSeq Reagent kit v3 (600-cycle) on a MiSeq sequencer (Illumina). Nanopore sequencing was conducted using a polymerase chain reaction-free rapid barcoding gDNA sequencing protocol (EXP-FLP002 and SQK-RBK004; Oxford Nanopore Technologies, UK) followed by sequencing using a FLO-MIN106D (R9.4.1) flow cell on a MinION Mk1B device. For Illumina sequencing, the raw reads obtained were trimmed and filtered using BBDuk (2), resulting in 1,386,852 reads of 104× coverage and a mean read length of 231 nt. For nanopore sequencing, following high accuracy mode base-calling with Dorado v.0.5.1 (3) and trimming using Porechop v.0.2.4 (4), a total of 204,860 reads of 317× coverage and N50 >4,774 bp were achieved. Illumina and nanopore reads were hybrid-assembled by first using Flye v.2.9.1 (5) to generate long-read contigs, then with Unicycler v.0.4.4 (6) to combine the contigs using both short- and long-reads. Gene prediction and annotation were performed using Prodigal v.2.6.3 (7) and PGAP v.6.9 (8). Antimicrobial resistance (AMR) genes were identified using ResFinder with database v.3.2 (9). Plasmids were identified and typed by using the mob_typer command within MOBsuite v.3.1.9 (10), and prophage sequences were analyzed using PHASTER v.2020-12-22 (11). Virulence, multilocus sequence typing, and PCR-serogroup were investigated using the BIGSdb database v.1.50.2 for *Listeria* (https://bigsdb.pasteur.fr/listeria/) (12). Default parameters were used for all of the bioinformatics tools except where otherwise noted.

The complete genome sequence of *L. monocytogenes* CFIAFB20240043 has a length of 3,080,806 bp with a total GC content of 37.9%. It contains a single circular chromosome and an 86 kb plasmid that is predicted to be conjugative based on the presence of a relaxase (MobP-type) and a mate-pair formation marker. Three intact prophage sequences of various sizes (21 kb, 52.4 kb, and 41.9 kb, with PHASTER scores of 110, 150, and 150, respectively) were identified from different chromosomal regions. Annotation reveals 3,056 coding sequences, 67 tRNAs, and 18 rRNAs. This strain was found to belong to PCR-serogroup: IIa (4), lineage: II, sequence type: 8, and clonal complex: CC8. The AMR gene *fosX* with 98.76% identity was found on the chromosome, in addition to 56 virulence-related genes. A classic *prfA*-regulated virulence gene cluster, also known as *Listeria* pathogenicity island 1, together with an intact *inlA* allele free of premature stop codon, was also observed.

## Data Availability

The complete genome sequence of *L. monocytogenes* strain CFIAFB20240043 was deposited in GenBank (accession numbers CP182819:CP182820). Base-called MinION and MiSeq reads in fastq format are available from the NCBI Sequence Read Archive (SRA) under accession numbers SRR32467288 and SRR32467287, respectively.
